# Non-Opsonic Phagocytosis of *Legionella pneumophila* by Macrophages Is Mediated by Phosphatidylinositol 3-Kinase

**DOI:** 10.1371/journal.pone.0003324

**Published:** 2008-10-02

**Authors:** Souvenir D. Tachado, Mustapha M. Samrakandi, Jeffrey D. Cirillo

**Affiliations:** Department of Microbial and Molecular Pathogenesis, Texas A&M Health Science Center, College Station, Texas, United States of America; Oregon Health & Science University, United States of America

## Abstract

**Background:**

*Legionella pneumophila*, is an intracellular pathogen that causes Legionnaires' disease in humans, a potentially lethal pneumonia. *L. pneumophila* has the ability to enter and replicate in the host and is essential for pathogenesis.

**Methodology/Principal Findings:**

Phagocytosis was measured by cell invasion assays. Construction of PI3K mutant by PCR cloning and expression of dominant negative mutant was detected by Western blot. PI3K activity was measured by ^32^P labeling and detection of phospholipids products by thin layer chromatography. Infection of macrophages with virulent *L. pneumophila* stimulated the formation of phosphatidylinositol 3-phosphate (PIP3), a phosphorylated lipid product of PI3K whereas two structurally distinct phosphatidylinositol 3 kinase (PI3K) inhibitors, wortmannin and LY294002, reduced *L. pneumophila* entry into macrophages in a dose-dependent fashion. Furthermore, PI3K activation led to Akt stimulation, a serine/threonine kinase, which was also inhibited by wortmannin and LY294002. In contrast, PI3K and protein kinase B (PKB/Akt) activities were lower in macrophages infected with an avirulent bacterial strain. Only virulent *L. pneumophila* increased lipid kinase activity present in immunoprecipitates of the p85α subunit of class I PI3K and tyrosine phosphorylated proteins. In addition, macrophages expressing a specific dominant negative mutant of PI3K reduced *L. pneumophila* entry into these cells.

**Conclusion/Significance:**

Entry of *L. pneumophila* is mediated by PI3K/Akt signaling pathway. These results suggest an important role for PI3K and Akt in the *L. pneumophila* infection process. They point to possible novel strategies for undermining *L. pneumophila* host uptake and reducing pathogenesis of Legionnaires' disease.

## Introduction

Legionnaire's disease is a severe bacterial infection of the respiratory tract caused by *Legionella pneumophila*. The disease was named for the 29 Legionnaires killed by mysterious pneumonia at an American Legion Convention in Philadelphia in 1976 [1]. The cause of this mysterious pneumonia was identified to be *Legionella pneumophila*, a bacteria that was proliferating in the hotel's air-conditioning system [2]. *L. pneumophila* is a Gram-negative bacterium that replicates almost exclusively intracellularly during infection [3], and in its natural freshwater habitat [4]. Macrophages are the primary cell type that *L. pneumophila* replicates within during infection [5], and has been shown to enter monocytes in an unusual mechanism called coiling phagocytosis [6].

Phagocytosis is a complex cellular function to both lower and higher organisms. Lower organisms such as slime molds and protozoa utilize this response primarily for acquisition of nutrients; while in higher organisms it is a critical component of the immune system, primarily through “professional” phagocytes such as macrophages, dendritic cells, monocytes and neutrophils [7]. Phagocytes remove and degrade cellular debris, foreign particles, apoptotic cells and potential infectious agents [8]. Changes in the activation status of components in the signal transduction cascades that affect phagocytosis can modulate this response. For example, such an association exists between changes in phosphoinositide-specific phospholipase C (PI-PLC) [9], protein kinase C (PKC) [10], phosphatidylinositol 3-kinase (PI3K) [11] and Rho GTPases [12] can also affect subsequent signal transduction events.

Pathogen mediated PI3K activation, in particular, has been implicated as being essential to phagocytosis induction [13]–[16]. One downstream consequence of PI3K activation is Akt or protein kinase B, stimulation [17]. Akt is activated following its recruitment from the cytoplasm to the cellular membranes. This occurs following PI3K interaction with the pleckstrin homology domain of PI3,4,5P_3_ and PI3,4P_2_ and result from PI3K mediated phosphorylation of PIP_2_ [18]. Signaling mediated increases in multiple PI3K lipid products are probably required to induce cell survival by mediating membrane fusion events leading to phagocytosis. Control of PI3K activity is implicated to be essential in the chain of signaling events linked to tyrosine-kinase receptor activation leading to actin polymerization and phagocytosis [19], [20].

The downstream signaling events occurring subsequent to entry of *L. pneumophila* into human monocytes [21], epithelial cells [22] and murine macrophages [23] are not fully described. It has been shown that actin polymerization is triggered by *L. pneumophila* during invasion of human monocytes [21], [24] and fibroblasts [22], but host cell protein(s) that control phosphorylation and actin polymerization during entry have not been identified. In the current study, we sought to identify proteins that control phosphorylation in macrophages during phagocytosis of *L. pneumophila*.

In the current study, we probed for PI3K pathway involvement in mediating phagocytosis subsequent to infection by *L. pneumophila* of human monocytes. Non-opsonic-mediated events in the lungs is relevant because the amount of serum in the lungs is minimal [25] and alveolar macrophages presumably lack receptor expression for serum opsonins [26]. We observed PI3K and Akt activation following *L. pneumophila* infection. Moreover, wortmannin and LY294002 inhibited invasion in a dose-dependent manner. Finally, invasion of *L. pneumophila* into macrophages was inhibited in macrophages expressing a dominant negative PI3K gene. These results suggest an important role for PI3K and Akt in the *L. pneumophila* infection process. They point to possible novel strategies for undermining *L. pneumophila* host uptake and reducing pathogenesis of Legionnaires' disease.

## Results

### PI3K inhibitors reduce non-opsonic phagocytosis of *L. pneumophila* by macrophages

**Figure 1:**
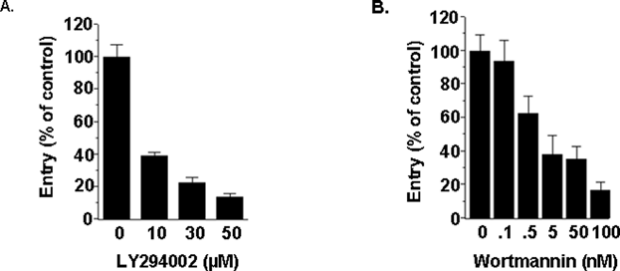
PI3K inhibitors block *L. pneumophila* entry into host cells. Murine J774A.1 (A, B) that were treated or not treated with different concentrations of LY294002 or (A) Wortmannin (B) for 30 min and subsequently infected with *L.pneumophila* for 1 hr. Entry in the absence of the inhibitor was arbitrarily set to 100%. Data presented are means+/−SEM for assays done in triplicate. Similar results were obtained in at least two independent experiments.

To determine whether PI3K pathway is involved in non-opsonic entry of *L. pneumophila* by macrophages, we tested the effects of two structurally unrelated compounds, wortmannin and LY294002, both of which specifically inhibit PI3K activity. Cultures of J774A.1 macrophages were treated with different concentrations of wortmannin and LY294002 for 30 min, followed by co-incubation with *L. pneumophila* (MOI 100∶1). As shown in Fig. 1A, entry of *L. pneumophila* into macrophages was inhibited by LY294002 in a dose-dependent fashion. Significant inhibition started at 10 µM and became maximal at 50 µM (p<0.01). Similar results were observed when macrophages were treated with different concentrations of wortmannin, a structurally unrelated PI3K inhibitor. Significant inhibition started at 0.5 nM (p<0.05) and reached a maximum value at 100 nM. Moreover, wortmannin was more effective in inhibiting *L. pneumophila* entry into macrophages compared to LY294002. The former inhibited entry with an IC_50_ of ∼2.5 nM, while the latter inhibited with an IC_50_ of 8.0 µM. These results are similar to those reported elsewhere [27], [28]. Furthermore, IC_50_ values of this study were similar to those reported for PI3K inhibition but not PI 4-kinase [27], and MLCK [29]. Similar results were obtained using primary human macrophages differentiated from peripheral blood monocytes (data not shown). These studies suggest that non-opsonic entry of *L. pneumophila* by murine macrophages is PI3K-dependent, as has been shown in several other microorganisms [9], [15], [30].

### Expression of a dominant negative mutant of p85α inhibits *L. pneumophila* entry

**Figure 2:**
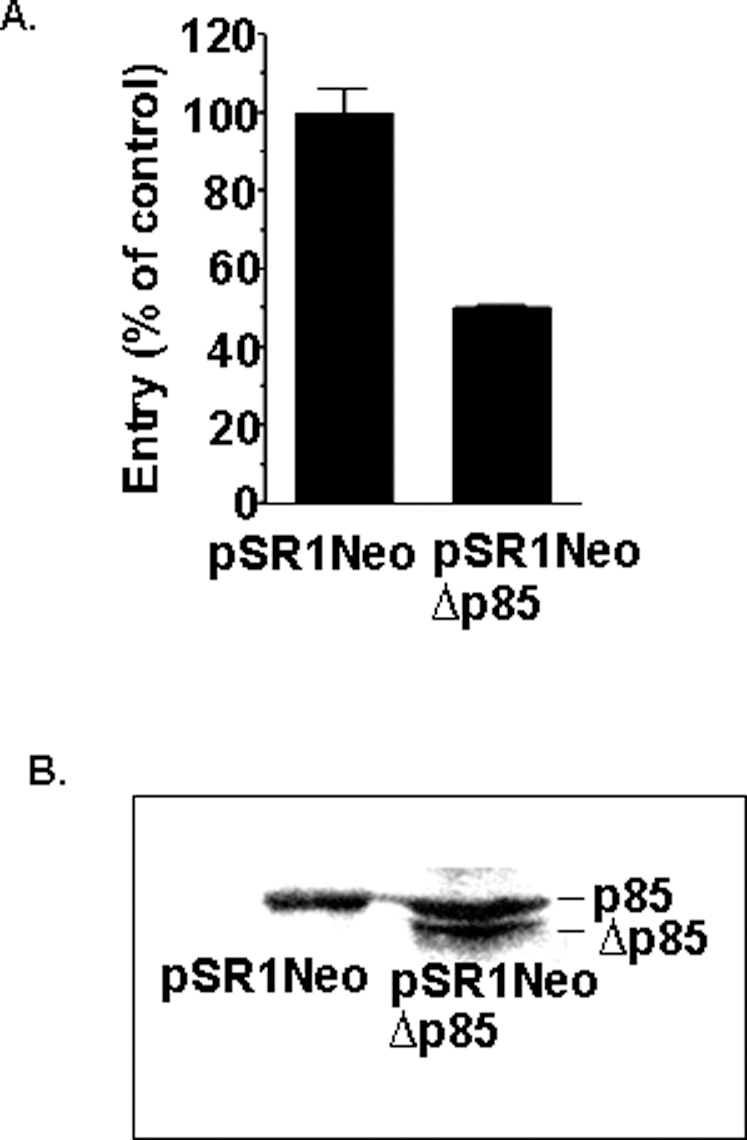
Overexpression of PI3K mutant protein ablates *L. pneumophila* entry. Entry by *L. pneumophila* into J774A.1 macrophages expressing the p85α mutant PI3K (pSR1NeoΔp85) and containing vector alone (pSR1Neo) after 1 hour co-incubation (A). Western analysis using antibody against the PI3K p85 α subunit, demonstrating Δp85 expression in transfected macrophages (B). Macrophage lysates were immunoprecipitated with anti-p85α and run on SDS/PAGE followed by Western blot analysis. Entry into macrophages carrying the vector alone was arbitrarily set to 100%. Data are the means+/−SEM for assays done in duplicate. Similar results were obtained in at least two independent experiments.

To confirm our pharmacological data regarding wortmannin and LY294002 phagocytosis inhibition, J774A.1 cells were transiently transfected with a dominant negative PI3K mutant gene (J774A.1∶Δp85) or empty vector. Western blot analysis of the cells transfected with the dominant negative p85 mutant and the empty vector confirm Δp85 expression in these macrophages (Fig. 2A). The protein encoded by this gene lacks amino acids 479–513 located between the two *src*-homology 2 (SH2) domains. This region is necessary and sufficient for interaction of the p85 subunit with the N terminus of p110 catalytic subunit, required for PI3K activity [31]. The ability of *L. pneumophila* to enter J774A.1∶Δp85 cells were reduced 50% as compared to normal J774A.1 cells carrying the empty vector (Fig. 2B). Even though this inhibitory effect resulting from dominant negative p85 expression is less than that obtained following drug exposure, it supports the notion that the PI3K pathway mediates *L. pneumophila* host uptake. The smaller decline could be due to the partial suppression of PI3K activity owing to lack of complete replacement of wild type functional p85 protein with its mutant counterpart. In addition, these differences could be due to inhibition of other cellular signaling pathways by Wortmannin such as PKC-ζ [32], DNA-dependent protein kinase (DNA-PK) [33] also involved in phagocytosis. These studies confirm the pharmacological data and support the involvement of PI3K in the ability of *L. pneumophila* to infect macrophages.

### *L. pneumophila* infection induces the activation of protein kinase B (Akt)

**Figure 3:**
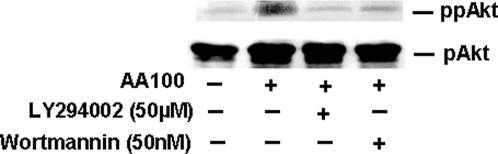
L. pneumophila infection stimulates PI3K-dependent phosphorylation of Akt. Macrophages were treated or not treated with PI3K inhibitors and infected with *L. pneumophila* for 15 min. Lysates were prepared and Ser^473^ phospho Akt (activated) detected by Western blot analysis (ppAkt). The membrane was stripped and re-probed with antibody against total cellular Akt, which is normally phosphorylated at a single position (pAkt). Similar results were obtained in at least two independent experiments.

To further demonstrate the involvement of PI3K in mediating phagocytosis of *L. pneumophila* by macrophages, we tested whether protein kinase B (Akt) is activated during phagocytosis. Akt mediates the effects of PI3K downstream in the PI3K signaling pathway. We utilized Western blot analysis with antibodies specific to the activated (phosphorylated) form of Akt to determine the levels of activated Akt in *L. pneumophila* infected macrophages. Macrophages were pre-treated in the presence or absence of PI3K inhibitors for 1 hr followed by *L. pneumophila* (MOI 100∶1) infection for 15 min and lysates prepared. Western blots were probed with an antibody recognizing Akt phosphorylated Ser^473^ (Cell Signaling, Beverly, MA). Infection of macrophages by *L. pneumophila* significantly elevated the level of Ser^473^ phosphorylated Akt as compared to the control, suggesting that Akt is activated during *L. pneumophila* infection (Fig. 3). Activation of Akt was blocked by pretreatment with Wortmannin. In addition, a second PI3K inhibitor LY294002 reduced the amount of Ser^473^ phosphorylated Akt to nearly basal levels. These data indicate that *L. pneumophila* infection of macrophages activates Akt which mediates the effects of PI3K distal in the signaling pathway.

### Recruitment of the p85α subunit of PI3K to a complex containing tyrosine-phosphorylated proteins correlates with virulence

**Figure 4:**
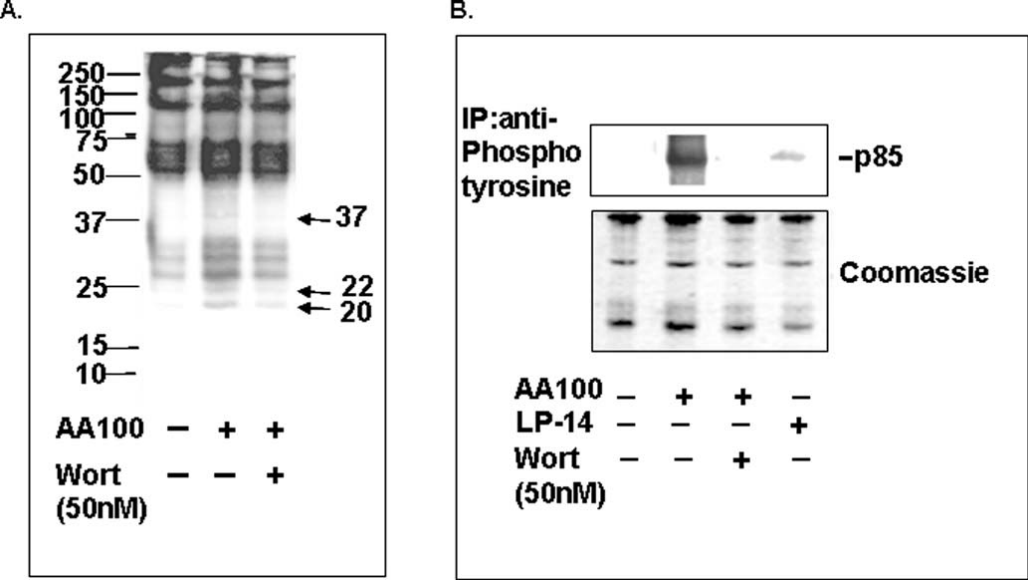
Virulent but not avirulent *L. pneumophila* induce association of PI3K with tyrosine-phosphorylated proteins. Macrophages were infected with the virulent (AA100) and avirulent (Lp-14) strains of *L. pneumophila* for 15 min. Lysates were then immunoprecipitated with anti-p85 antibody and probed with anti-phosphotyrosine antibody (A) or co-immunoprecipitated with anti-phosphotyrosine antibody and probed with anti-p85 (B). The levels of total protein present in each immunoprecipitate were similar as judged by Coomassie staining of identical gels run in parallel (lower panel). Similar results were obtained in at least two independent experiments.

Recruitment of the p85/p110-type PI3K to the plasma membrane and its association with tyrosine phosphorylated proteins occurs following stimulation is a key event in promoting its activation [11]. Tyrosine phosphorylation and activation of PI3K in *Trypanosoma cruzi* [9], *Listeria monocytogenes* [30] and in *Cryptosporidium parvum* [15], have been shown to play a key role in host cell invasion. A role for tyrosine phosphorylation in *L. pneumophila* entry into host cells has been previously observed [21]–[23]. We hypothesized that virulence depends on these tyrosine phosphorylation events mediating activation of p85/p110-type PI3K. It was tested by infecting macrophages with virulent (AA100) and avirulent Lp-14 *L. pneumophila* and analyzing for PI3K activity. We tested whether tyrosine phosphorylated proteins are induced by *L. pneumophila*, and whether PI3K activity is associated with this response. Infection of macrophages by *L. pneumophila* consistently increased phosphorylation of several major components migrating with apparent molecular weights (Mr) of 27, 30, and 35 kDa (Fig. 4A). Importantly proteins migrating with apparent molecular weights of 20, 22, and 37 KDa are *de novo* proteins and inhibited by wortmannin suggesting that phosphorylation is mediated by PI3K.

Next, we tested for an interaction between tyrosine phosphorylated proteins and PI3K. Macrophages were infected with *L. pneumophila* and lysates were collected and immunoprecipitated with anti-phosphotyrosine antibody followed by Western blotting. Membranes were probed with anti-p85 antibody. The PI3K subunit p85α associated with tyrosine phosphorylated proteins is nearly undetectable in uninfected macrophages, while its levels are greatly increased after *L. pneumophila* infection (Fig. 4B). Additionally, the presence of p85 in this protein complex can be inhibited by wortmannin. Interestingly, the avirulent *L. pneumophila* strain Lp-14 did not significantly increase the levels of p85 present in a complex that co-precipitates with tyrosine phosphorylated proteins (Fig. 4B). Taken together, these data suggest that *L. pneumophila* entry into macrophages induces tyrosine phosphorylation of proteins that recruit and activate PI3K. These data are consistent with other studies demonstrating recruitment of p85 into the plasma membrane by phosphorylated protein tyrosine kinases during invasion of mammalian cells by protozoa [9] and bacteria [30].

### Virulent but not avirulent strains of *L. pneumophila* stimulate PI3K activity

**Figure 5:**
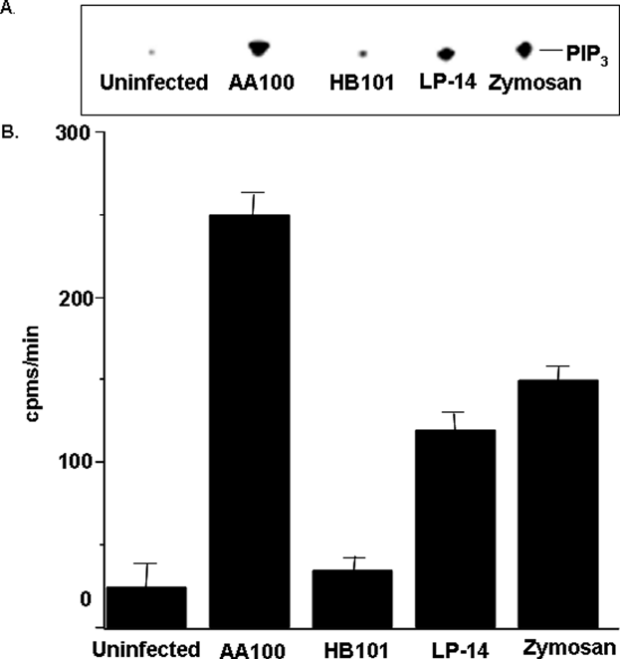
Virulent but not avirulent strains of *L. pneumophila* stimulate PI3K activity. Thin layer chromatography of lipids from *in vitro* kinase assays on infected macrophage lysates (A). Macrophages were stimulated with different ligands for 15 min. Lysates were immunoprecipitated with specific antiserum to p85^α^, and immune complexes were subjected to *in vitro* kinase assays using exogenous ATP-γ^32^P and PI as substrates followed by fluorography. The line (PIP) denotes the position of PI3P standard. Spots aligning at the same position as the PI3P standard were scraped and counted using scintillation counter (B). Similar results were obtained in at least two independent experiments.

We examined the ability of avirulent strain Lp-14 to stimulate PI3K as compared to wild-type virulent *L. pneumophila*. Strain Lp-14 is an AA100 mutant that is sodium resistant and unable to replicate in monocytic cells [34]. In addition, we tested other ligands in these assays, including non-pathogenic *E. coli* (HB101) and the yeast cell wall extract zymosan. The virulent strain *L. pneumophila* activated PI3K 10-fold, as measured by the levels of phosphoinositide 3-phosphates (PIP_3_) obtained from extracts using phosphatidylinositol (PI) as a substrate (Fig. 5). In contrast, Lp-14 activated PI3K four-fold compared to that of the control, suggesting that there is a correlation between virulence and the degree of activation of PI3K. PI3K activity is barely detectable in uninfected macrophages and macrophages infected with non-pathogenic *E. coli*. Zymosan activates PI3K at levels comparable to that of Lp-14. These data suggest that induction of PI3K during *L. pneumophila* entry into macrophages correlates with virulence.

## Discussion

The data presented in the current study indicate a role for host signal transduction processes involving tyrosine kinases PI3K and Akt during entry into murine macrophages. PI3K and Akt activation is implicated since *L. pneumophila* entry into macrophages is inhibited by two structurally distinct inhibitors of PI3K activity, wortmannin and LY294002, in a dose-dependent fashion. Wortmannin has previously been shown to inhibit entry into host cells by other pathogenic microorganisms, including *Cryptosporidium parvum* [15], *T. cruzi* [35] and *L. monocytogenes* [30]. The levels of these inhibitors required to inhibit entry into macrophages by *L. pneumophila* are low enough that they should be relatively specific to PI3K activity, particularly because this effect is dose-dependent [36]. These results contradict those obtained in an earlier report wherein phagocytosis of wild-type *L. pneumophila* by U937 cells was not inhibited by wortmannin, whereas avirulent strain (25D) intake was suppressed [24]. The reasons for these seemingly conflicting results remain unclear; however, there are a number of differences in the experimental design that could be responsible. First, in the current study we used *L. pneumophila* strain AA100; whereas, the previous study used strain JR32 and there are several genetic and phenotypic differences between these strains that can impact on the efficiency of entry and virulence [37]. Since strain AA100 enters macrophages at higher levels than strain JR32, subtle differences in the efficiency of entry may be more readily observed in strain AA100 and since this strain is more virulent in mice, these differences may be relevant to pathogenesis. Second, different host cells were used in these studies, making it difficult to compare the results obtained. Irregardless, we have confirmed the effects we have observed with several complementary experimental strategies and our data are not solely dependent upon inhibition of phagocytosis, but also on observation of specific differences in signal transduction.

Use of pharmacological inhibitors in biological assays has been supplanted in recent years by transfer of gene of interest in the form that confers loss or gain of function. This approach is preferred because it circumvents inherent problems of specificity using pharmacological inhibitors. To confirm our entry assays with PI3K inhibitors, we evaluated entry of *L. pneumophila* into macrophages expressing a dominant negative mutant of PI3K. *L. pneumophila* entry into macrophages was significantly lower in macrophages transfected with the pSR1αNeoΔp85 as compared to cells transfected with pSR1αNeo alone. The number of internalized bacteria in macrophages transfected with pSR1αNeo is comparable to those internalizing an empty vector suggesting that the presence of the plasmid does not affect entry (data not shown). Taken together, these results support a role for PI3K during infection of macrophages by *L. pneumophila*, similar to several other intracellular pathogens [9], [30], [38].

Possibly, the PI3K/Akt signal transduction pathway serves as a pathway utilized by a number of intracellular pathogens that must enter and survive within host cells. Examples of key steps resulting from interactions of host cells with pathogens include MAP kinases [39], tyrosine kinases [40], p70 S6 kinases [41] and phospholipase D activation [42]. It has been shown that contact between host cells and *L. pneumophila* induces host tyrosine phosphorylation [21]–[23] and actin polymerization [21], [24]. These signaling events have been implicated in phagocytosis of *L. pneumophila* by host cells. However, the protein(s) that control each of these events are not well defined. The current study suggests the involvement of PI3K in mediating non-opsonic phagocytosis of *Legionella*. Previous data indicate that activation of Rac1 and Rho, members of Rho-family GTPases, requires PI3K activity, which in turn activates p21-activated kinase-1 [43], and Rho-kinases [44] to control cytoskeletal rearrangements. Thus, this pathway may well play an important role in phagocytic events.

We have shown that contact between *L. pneumophila* and macrophages induce activation of Akt. One of the responses to Akt activation is prevention of programmed cell death or apoptosis [45]. Prolonging the survival of infected cells is clearly in the best interest of *L. pneumophila,* as it will allow optimal replication and dissemination of the bacteria in the host. Thus, further investigation of the effects of Akt activation on phagocytosis as well as subsequent intracellular survival and replication of *L. pneumophila* could provide useful information regarding the intricacies of these interactions.

Cell entry of *T. cruzi* promotes PI3K recruitment to the plasma membrane leading to its association with tyrosine-phosphorylated proteins which can be followed by PI3K activation [9], and *L. monocytogenes* [30], However, it is also tenable that activation of PI3K results in tyrosine phosphorylation of other non membrane localized proteins, which then recruit PI3K. Even though our data show that entry of *L. pneumophila* into macrophages induces the recruitment of PI3K to a complex of tyrosine phosphorylated proteins, it is not possible to distinguish between the two aforementioned alternatives.

Our findings support the hypothesis that PI3K activation is required for efficient entry of *L. pneumophila* into macrophages. Activation of PI3K leads to the formation of 3-phosphoinositides and activation of Akt. Further studies to determine the isoform(s) of PI3K involved would be useful, since this information will provide insight into the mechanisms of PI3K activation. The conclusion that PI3K is involved in uptake of *L. pneumophila* into macrophages is based on several lines of evidence: (i) two distinct PI3K inhibitors, wortmannin and LY294002 can specifically block uptake of *L. pneumophila* by macrophages in a dose-dependent manner; (ii) virulent *L. pneumophila* induces greater PI3K activity and recruitment of PI3K to a complex of tyrosine phosphorylated proteins than an avirulent strain; (iii) infection induces the activation of Akt and this activation is dependent on PI3K; (iv) infection increases recruitment of PI3K to a complex of tyrosine phosphorylated proteins and this recruitment is dependent on PI3K; and (v) macrophages expressing a mutant PI3K display reduced *L. pneumophila* uptake. Collectively these results indicate that *L. pneumophila* uptake can be triggered by activation of PI3K resulting from an intricate interplay between the pathogen and the host cells.

## Materials and Methods

### Reagents

Anti-phosphotyrosine mouse monoclonal antibody 4G10 was purchased from Upstate Biotechnology, Inc. (Lake Placid, NY); anti-p85α from Santa Cruz; protein A/G sepharose beads from BioRad; Wortmannin, LY294002 and zymosan from ICN Biomedical Inc.; anti-phospho Akt and total Akt from New England Biolabs; genistein, protease inhibitor cocktail and PMSF from Sigma; and ECL from Amersham.

#### Bacterial strains and growth conditions

The *L. pneumophila* strain used for these studies was the streptomycin-resistant variant [46] of *L. pneumophila* serogroup 1 strain AA100 [47]. This strain has been shown to be virulent in both *in vitro* and *in vivo* models of infection and was passaged no more than twice in the laboratory before use to avoid loss of virulence. The strain Ψlp55 (Lp-14) is an AA100 clone that has been passed fourteen times in the laboratory until it is sodium resistant and unable to replicate in monocytic cells [34]. *L. pneumophila* were grown on buffered charcoal yeast agar (BCYE) agar for three days at 37°C in 5% CO_2_.

#### Cell culture

The murine macrophage-like cell line J774A.1 was from American Type Culture Collection (ATCC #TIB 67). J774A.1 cells were grown in Dulbecco's modified Eagle's medium (DMEM); BioWhittaker, Walkersville, MD) supplemented with 10% heat-inactivated fetal calf serum (HyClone, Logan, UT) and 4 mM L-glutamine (BioWhittaker).

#### Cell invasion assays

The effects of PI3K inhibitors on *L. pneumophila* phagocytosis were examined as described previously [48]. Briefly, macrophages were plated at a density of 2.5×10^5^ per well on 24- well culture plates (Costar, Cambridge, MA) and allowed to adhere overnight at 37°C in a humidified atmosphere containing 5% CO_2_. The next day, cells were suspended in fresh medium and treated with different concentrations of PI3K inhibitors (0.1–100 nM for Wortmannin and 10–50 µM for LY294002) for 30 min. Cells were then co-incubated with *L. pneumophila* at a multiplicity of infection (MOI) of 100 and incubated for 1 hr at 37°C in a humidified atmosphere containing 5% CO_2_. Extracellular bacteria were removed by washing the cells with PBS and incubated in media containing 100 µg/ml of gentamicin (Sigma) for 1 hr at 37°C in 5%CO_2_. Cells were washed three times with PBS and lysed in 1 ml of sterile water for 10 min at room temperature. The number of intracellular bacteria was determined by plating appropriate dilutions of lysates on BCYE incubated at 37°C in a humidified atmosphere containing 5% CO_2_. The percent invasion for bacterium-infected cells that became gentamicin resistant that were not treated with inhibitors (control) was normalized to 100%. The percent invasion of bacterium-infected cells in the presence of inhibitors was calculated by dividing the number of CFUs per milliliter of these samples by the number of CFUs per milliliter of the control, multiplied by 100. To correct for variation in the levels of uptake between experiments, entry was reported relative to *L. pneumophila* AA100 (i.e. relative entry = % entry of test strain/% entry of AA100).

#### Construction of a PI3K mutant macrophage cell line

Deletion of the active site of the bovine subunit p85α [49] of PI3K was constructed previously by PCR cloning from the p85α cDNA [50]. The resulting construct (pGEX-2TΔp85) expresses a Δp85α mutant protein that has a deletion of 35 amino acids (a.a. 478–513) replaced by two amino acids, serine and arginine [50]. The fragment Δp85α carried by pGEX-2TΔp85α was gel-purified after digestion of this construct by *BamH*I and *EcoR*I enzymes and cloned (after fill-in) into *Eco*RV of the multiple cloning site of pSRα1Neo mammalian expression vector (D. Wettstein and M. Davis, Stanford University). J774A.1 cells (5×10^5^/well) were transfected with 2 µg of plasmid using Lipofectamine (Invitrogen) in serum-free DMEM following manufacturer's instructions. At 3 h post-transfection cells were suspended in serum containing medium. At 24–48 h post-transfection, invasion assays were carried out and Western blot analysis was used to monitor p85α expression. Invasion assays were carried out in parallel using cells containing pSRα1NeoΔp85 and the vector alone.

#### Western blot analyses

Western blot analysis was performed as previously described [51]. Cells were incubated at 37°C in the presence or absence of inhibitors for 30 min. Cells were then infected with *L. pneumophila* at an MOI of 100, and incubated for an additional 15 min. Cells were washed three times with PBS containing 1 mM sodium orthovanadate plus 5 mM EDTA and scraped into lysis buffer containing 20 mM Tris-HCl (pH 8.0), 1% Triton X-100, 10% glycerol, 100 mM NaCl, 2 mM EDTA, 1 µM PMSF, 20 µM leupeptin, and 0.15 U/ml aprotinin. Samples were placed on ice for 30 min and triton X-100 soluble proteins isolated by centrifugation (10,000×g for 15 min at 4°C). Sample buffer containing 62.5 mM Tris-HCl (pH 6.8), 2.3% SDS, 100 mM DTT, and 0.005% bromphenol blue was added to the cell lysates and boiled for 5 min. 10 µg of each extract were subjected to SDS-PAGE and electrophoretically transferred to nitrocellulose membranes. Western analysis was performed on the nitrocellulose membranes by adding the appropriate primary antibody followed by the use of horseradish peroxidase-conjugated anti-mouse Ig as the secondary antibody. ECL reagents (Amersham) were used for detection of peroxidase activity.

#### Immunoprecipitation

Immunoprecipitation was performed as previously described [52]. Briefly, cells were lysed in ice-cold buffer containing 50 mM Tris-HCl, pH 7.5, 150 mM NaCl, 2 mM EDTA, 0.1% Tween 20, 0.1% SDS, 10 µg/ml aprotinin, 10 µg/ml leupeptin, 5 mM NaF, 1 mM phenylmethylsulfonyl fluoride, 4 mM Na_3_VO_4_ for 30 min. Cellular debris were removed by centrifugation at 13,000 rpm for 15 min and detergent lysates were pre-cleared with pre-immune serum, and protein concentration determined using the Bradford method [53]. Appropriate antibodies were then added to the pre-cleared cell lysates, incubated overnight at 4°C with constant mixing. Protein A/G sepharose was added and the reaction mixtures were further incubated at 4°C for 2 hr with constant mixing. Immunoprecipitated proteins were then subjected to SDS/PAGE and Western blot analysis.

#### Measurement of PI3K activity

Measurement of PI3K activity was carried out in a similar manner to that previously described [9]. Briefly, cell lysates were immunoprecipitated with anti-phosphotyrosine (4G10) and incubated overnight with constant mixing. The antigen-antibody complexes were captured by the addition of protein A/G sepharose and incubated for 2 hr at 4°C. Immunoprecipitated proteins were washed three times with ice-cold 1% Nonidet P-40 in PBS, twice in buffer containing 10 mM Tris-HCl, pH 7.5, 100 mM Na_3_VO_4_, 100 mM NaCl, 1 mM EDTA, and once in PI3K buffer (20 mM Tris-HCl pH 7.5, 100 mM NaCl, 0.1 mM EGTA, 10 mM MgCl_2_). The immunoprecipitate was then suspended in 0.16 ml of PI3K buffer containing 200 µg of sonicated phosphatidylinositol and 10.0 µCi of γ-^32^P] ATP. The reaction mixture was incubated for 20 min at room temperature and the reaction stopped by addition of 100 µl of 1 M HCl followed by the addition of 200 µl of chloroform: methanol (1∶1, vol∶vol). The reaction mixture was spun at 10,000 rpm, and the organic phase spotted onto silica TLC G60 plates (20×20×250 µm), precoated with 1% potassium oxalate. Plates were developed using chloroform: methanol: H20∶NH4OH (60∶47∶11∶2, vol∶vol). The standards were visualized with iodine vapor. Radiolabeled bands were located by autoradiography or phosphor imaging, and the PIP band was scraped into scintillation vials and the associated radioactivity determined using a scintillation counter.
